# TIF1γ interferes with TGFβ1/SMAD4 signaling to promote poor outcome in operable breast cancer patients

**DOI:** 10.1186/s12885-015-1471-y

**Published:** 2015-06-04

**Authors:** Loay Kassem, Mathieu Deygas, Laurent Fattet, Jonathan Lopez, Thibaut Goulvent, Emilie Lavergne, Sylvie Chabaud, Nicolas Carrabin, Nicolas Chopin, Thomas Bachelot, Germain Gillet, Isabelle Treilleux, Ruth Rimokh

**Affiliations:** 1 Clinical Oncology Department, Faculty of medicine, Cairo University, Cairo, Egypt; 2 U1052 Inserm, UMR CNRS 5286. Université de Lyon, Centre de Recherche en Cancérologie de Lyon, Centre Léon Bérard, Lyon, France; 3 Biostatistics Unit, Centre Léon Bérard, Lyon, France; 4 Department of surgery, Centre Léon Bérard, Lyon, France; 5 Department of medical oncology, Centre Léon Bérard, Lyon, France; 6 Department of pathology, Centre Léon Bérard, Lyon, France

## Abstract

**Background:**

The Transforming growth factor β (TGFβ) signaling has a paradoxical role in cancer development and outcome. Besides, the prognostic significance of the TGFβ1, SMAD4 in breast cancer patients is an area of many contradictions. The transcriptional intermediary factor 1γ (TIF1γ) is thought to interact with the TGFβ/SMAD signaling through different mechanisms. Our study aims to define the prognostic significance of TGFβ1, SMAD4 and TIF1γ expression in breast cancer patients and to detect possible interactions among those markers that might affect the outcome.

**Methods:**

Immunohistochemistry was performed on tissue microarray (TMA) blocks prepared from samples of 248 operable breast cancer patients who presented at Centre Léon Bérard (CLB) between 1998 and 2001. The intensity and the percentage of stained tumor cells were integrated into a single score (0–6) and a cutoff was defined for high or low expression for each marker. Correlation was done between TGFβ1, SMAD4 and TIF1γ expression with the clinico-pathologic parameters using Pearson’s chi-square test. Kaplan-Meier method was used to estimate distant metastasis free survival (DMFS), disease free survival (DFS) and overall survival (OS) and the difference between the groups was evaluated with log-rank test.

**Results:**

223 cases were assessable for TIF1γ, 204 for TGFβ1 and 173 for SMAD4. Median age at diagnosis was 55.8 years (range: 27 to 89 years). Tumors were larger than 20 mm in 49.2 % and 45.2 % had axillary lymph node (LN) metastasis (N1a to N3). 19.4 % of the patients had SBR grade I tumors, 46.8 % grade II tumors and 33.9 % grade III tumors. ER was positive in 85.4 %, PR in 75.5 % and Her2-neu was over-expressed in 10 % of the cases. Nuclear TIF1γ, cytoplasmic TGFβ1, nuclear and cytoplasmic SMAD4 stainings were high in 35.9 %, 30.4 %, 27.7 % and 52.6 % respectively. TIF1γ expression was associated with younger age (p = 0.006), higher SBR grade (p < 0.001), more ER negativity (p = 0.035), and tumors larger than 2 cm (p = 0.081), while TGFβ1 was not associated with any of the traditional prognostic factors.

TGFβ1 expression in tumor cells was a marker of poor prognosis regarding DMFS (HR = 2.28; 95 % CI: 1.4 to 3.8; p = 0.002), DFS (HR = 2.00; 95 % CI: 1.25 to 3.5; p = 0.005) and OS (HR = 1.89; 95 % CI: 1.04 to 3.43; p = 0.037). TIF1γ expression carried a tendency towards poorer DMFS (p = 0.091), DFS (p = 0.143) and OS (p = 0.091). In the multivariate analysis TGFβ1 remained an independent predictor of shorter DMFS, DFS and OS. Moreover, the prognostic significance of TGFβ1 was more obvious in the TIF1γ high patient subgroup than in the patients with TIF1γ low expression. The subgroup expressing both markers had the worst DMFS (HR = 3.2; 95 % CI: 1.7 to 5.9; p < 0.0001), DFS (HR = 3.02; 95 % CI: 1.6 to 5.6; p < 0.0001) and OS (HR = 2.7; 95 % CI: 1.4 to 5.4; p = 0.005).

**Conclusion:**

There is a crosstalk between the TIF1γ and the TGFβ1/SMAD4 signaling that deteriorates the outcome of operable breast cancer patients and when combined together they can serve as an effective prognostic tool for those patients.

**Electronic supplementary material:**

The online version of this article (doi:10.1186/s12885-015-1471-y) contains supplementary material, which is available to authorized users.

## Background

Transforming growth factor-beta (TGFβ) belongs to a superfamily of polypeptides that controls cell proliferation, differentiation, motility and apoptosis in different cell types [1]. TGFβ1, one of the 3 isoforms of TGFβ, is a potent negative regulator of mammary gland epithelial cell proliferation [2, 3]. Several studies have demonstrated that TGFβ1 regulates many steps of normal mammary gland development and plays an important role in breast carcinogenesis [1, 4].

TGFβ signaling was proven to have dual role in cancer development as it displays both tumorigenic and tumor-suppressive effects. In early stages of tumor development TGFβ signaling suppresses tumor formation by its anti-proliferative and anti-apoptotic effects and the loss of TGFβ signaling was found to be one of the drivers of breast malignancy initiation. On the other hand, in later stages of carcinogenesis TGFβ1 signaling promotes metastasis by promoting epithelial-to-mesenchymal transition (EMT), angiogenesis and immunosuppression [4–6].

TGFβ signaling is triggered by binding of TGFβ to its receptor with the dimerization of TGFβ type I and II receptors (TβRIIs) leading to phosphorylation of the receptor regulated (R-) SMAD2 and SMAD3. Phosphorylated SMADs combine with common mediator (co-) SMAD4 that migrates to the nucleus. The SMAD complexes interact with different transcription factors regulating several target genes that control proliferation, metabolism and migration of malignant cells [7] . Besides this classical TGFβ/SMAD signaling, other SMAD independent signaling pathways also exist such as activation of mitogen activated protein kinases (MAPK) [8].

The transcriptional intermediary factor 1γ (TIF1γ), is a ubiquitous nuclear protein that has been implicated in TGFβ signaling through its binding to phosphorylated Smad2/3 [9]. TIF1γ could also antagonize Smad4 through its ubiquitin ligase activity [10]. We have recently demonstrated that TIF1γ regulates the TGFβ-induced EMT in mammary epithelial cells and during terminal differentiation of mammary alveolar epithelial cells and lactation through repression of Smad4 activity [11–13]. Most of data from mouse models suggest a tumor suppressor role for TIF1γ [14–16]. However, a recent study has demonstrated that overexpression of TIF1γ occurs during the early stages of colorectal carcinogenesis, suggesting a role in promoting colorectal cancer [17].

Expression of the TGFβ pathway markers in breast cancer revealed highly contradictory results. On one hand several studies observed that higher TGFβ1 levels in tumors or in the blood of breast cancer patients could predict a better outcome and less distant metastases [18–20]. On the other hand, several other studies reported that TGFβ1 expression carries a poor prognosis in those patients [21, 22].

In this retrospective study we analyzed the pattern of expression of TGFβ1, SMAD4 and TIF1γ in breast cancer tumors. We further analyzed the prognostic significance of each marker and the effect of the interactions between those 3 key players of the TGFβ signaling pathway on the outcome of breast cancer patients that might explain the contradictory results from the literature.

## Methods

### Patient population

We screened 353 consecutive female patients with operable primary breast cancer who underwent radical surgery and received adjuvant/neoadjuvant therapy at Centre Léon Bérard (CLB) between January 1998 and December 2001. Paraffin blocks of tumor tissue were available for 320 patients. Among these, we failed to assess any of the biomarker staining in 61 tumor specimens as a result of insufficient tumor or tissue loss during tissue microarray (TMA) preparation. Therefore, specimens from 259 patients with operable primary breast cancer were analyzed in this study. 11 patients were not included in the analysis as they were discovered to have metastatic disease at the initial diagnosis. In the remaining 248 samples (due to further tissue loss during slide preparation), 223 cases were assessable for TIF1γ, 204 cases for TGFβ1 and 173 cases for SMAD4 expression. A flowchart of the whole population and subsets tested for different biomarkers is shown in Additional file 1: Figure S1.

Patients underwent either modified radical mastectomy, or breast-conserving surgery. Lymph node invasion was assessed by axillary sentinel node and/or level I and II lymph node dissection and the number of lymph nodes (LNs) harboring metastasis was determined based on histologic examination. Tumor size was defined as the maximum tumor diameter measured on the tumor specimens at the time of surgery. Estrogen receptors (ER) and progesterone receptors (PgR) were detected by immunohistochemistry and tumors were considered positive if they had a nuclear staining in 10 % or more of the tumor cells. Human epidermal growth factor receptor 2 (HER2) expression was determined using immunohistochemistry and tumors were considered positive if they had 3+ staining by immunohistochemistry or 2+ staining with Her-2 amplification detected by FISH.

The data exported from the patients’ files for analysis included: age, histologic subtype, maximum tumor size, number of LNs involved, SBR grade, ER, PgR expression, HER2 overexpression, date of diagnosis, date of relapse, date of death and date of last news. This study is reported according to the REMARK criteria [23] and was done according to French regulations and approved by the ethics committee of the Centre Léon Bérard.

### Immunohistochemical analysis

Breast tumour samples were inserted as triplicates using a 600 μm needle in 8 Tissue Micro Array (TMA) blocks. The blocks containing invasive carcinoma were sectioned at a thickness of 4 μm. After deparaffinization and rehydration, endogenous peroxidases were blocked by incubating the slides in 5 % hydrogen peroxide in sterile water. For heat induced antigen retrieval, tissue sections were boiled in 10 mM Citrate Buffer pH6 (Dako, Trappes, France) using a water bath at 98 °C for 50 minutes.

The slides were then incubated at room temperature for 60 minutes with the antibodies against TGFβ1 (mouse monoclonal antibody MCA797 clone TB21 from AbD Serotec, Munich, Germany), TIF1γ (mouse monoclonal antibody TIF3E9 clone from Euromedex), SMAD4 (mouse monoclonal antibody SC-7966 clone B-8 from Santa Cruz, TX, USA).

These antibodies were diluted using an antibody diluent solution (Chemmate, Dako, Trappes, France) at 1/100 (for anti-TGFβ1), 1/500 (for anti-TIF1γ), 1/250 (for anti-SMAD4). After rinsing in Phosphate Buffer Saline, the slides were incubated either with a biotinylated secondary antibody bound to a streptavidin peroxidase conjugate (LSAB+ Kit, Dako, Trappes, France) for anti-TGFβ1 and SMAD4 or with the Flex kit (Ref K800021-2, Dako, Trappes, France) for anti- TIF1γ antibody. Bound antibody was revealed by adding the substrate 3, 3-diamino-benzidine. Sections were counterstained with hematoxylin.

Blinded to the clinical data, the biomarkers expression was evaluated by 2 observers who assessed both the percentage and the intensity of cytoplasmic staining for TGFβ1 and SMAD4 and of nuclear staining for TIF1γ and SMAD4 in the infiltrative carcinomatous cells only. For scoring purposes, the highest intensity of staining in malignant cells was classified into 4 levels (0: no staining, 1: weak staining, 2: moderate staining, 3: strong staining) and the percentage of the stained cells was also classified into 4 levels (0: no stained cells, 1: staining in less than one third of the malignant cells, 2: staining in one to two thirds of the malignant cells, 3: staining in more than two thirds of the malignant cells). Then both the intensity and the percentage scores were added to conclude a single score (from 0 to 6) in a manner similar to the Allred score for ER and PR staining [24].

For the purpose of correlations and survival analyses, tumours were considered to have low expression of cytoplasmic TGFβ1, and nuclear or cytoplasmic SMAD4 if they had a score of 0–2 and were considered to have high expression if they had a score of more than 2, while tumours were considered as nuclear TIF1γ low if they had a score of 0–3 and were considered as TIF1γ high if they had a score more than 3. Choice of the cut-off for high or low biomarker expression was based on the most discriminative cut-off in terms of survival analysis. Finally, patients were considered to have total SMAD4 low (SMAD4 loss) if they had low or no expression of both nuclear and cytoplasmic SMAD4.

### Statistical analysis

The correlation between TIF1γ, TGFβ1, SMAD4 expression and clinico-pathologic characteristics, was determined using Pearson’s chi square test (or Fisher’s exact test) for categorical variables and Student’s T test for numerical variables. Disease-free survival (DFS) was defined as the time from the date of diagnosis of breast cancer to the date of any cancer recurrence (local or distant or contralateral), or death. Distant metastasis free survival (DMFS) was defined as the time from the date of diagnosis of breast cancer to the date of distant metastasis or death. Overall survival (OS) was defined as the time from the date of diagnosis of breast cancer to the date of death.

To account for the heterogeneous follow up period resulting from different dates of diagnoses and last follow up visits, we locked the database at a maximum of 12 years of follow up and patients without events at 12 years were censored.

Survival curves, median DMFS, DFS and OS (if reached) in addition to 8 year DMFS, DFS and OS rate (with 95 % CIs) were derived from Kaplan-Meier estimates and the curves were compared using log-rank test [25]. Hazard ratios and 95 % CIs were calculated using cox regression model [26]. Cox multivariate analysis was performed using cox regression model to determine whether a factor is an independent predictor of DMFS, DFS or OS after adjusting for other significant factors at the univariate level. All statistical tests were two-sided, and the p value was considered statistically significant if less than 5 %. The statistical analyses were performed using SPSS 17.0 statistics package (SPSS Inc, Chicago, IL).

## Results

### Clinico-pathological characteristics

For the 248 assessable patients the median follow up interval was 9.7 years (range: 2 m to 12y). Median age at diagnosis was 55.8 years (range: 27 to 89 years). 49.2 % had tumors larger than 20 mm, 45.2 % had LN metastasis. 19.4 % of the patients had SBR grade I tumors, 46.8 % grade II tumors and 33.9 % grade III tumors. ER was positive in 85.4 %, PR in 75.5 % and Her2-neu was over-expressed in 10 % of the cases. 56.5 % of the patients received adjuvant chemotherapy while 77.4 % received adjuvant hormonal therapy. Additional file 1: Table S1 shows the clinico-pathological characteristics of the tested patients' cohort.

### Expression profile of TIF1γ, TGFβ1 and SMAD4

**Fig. 1:**
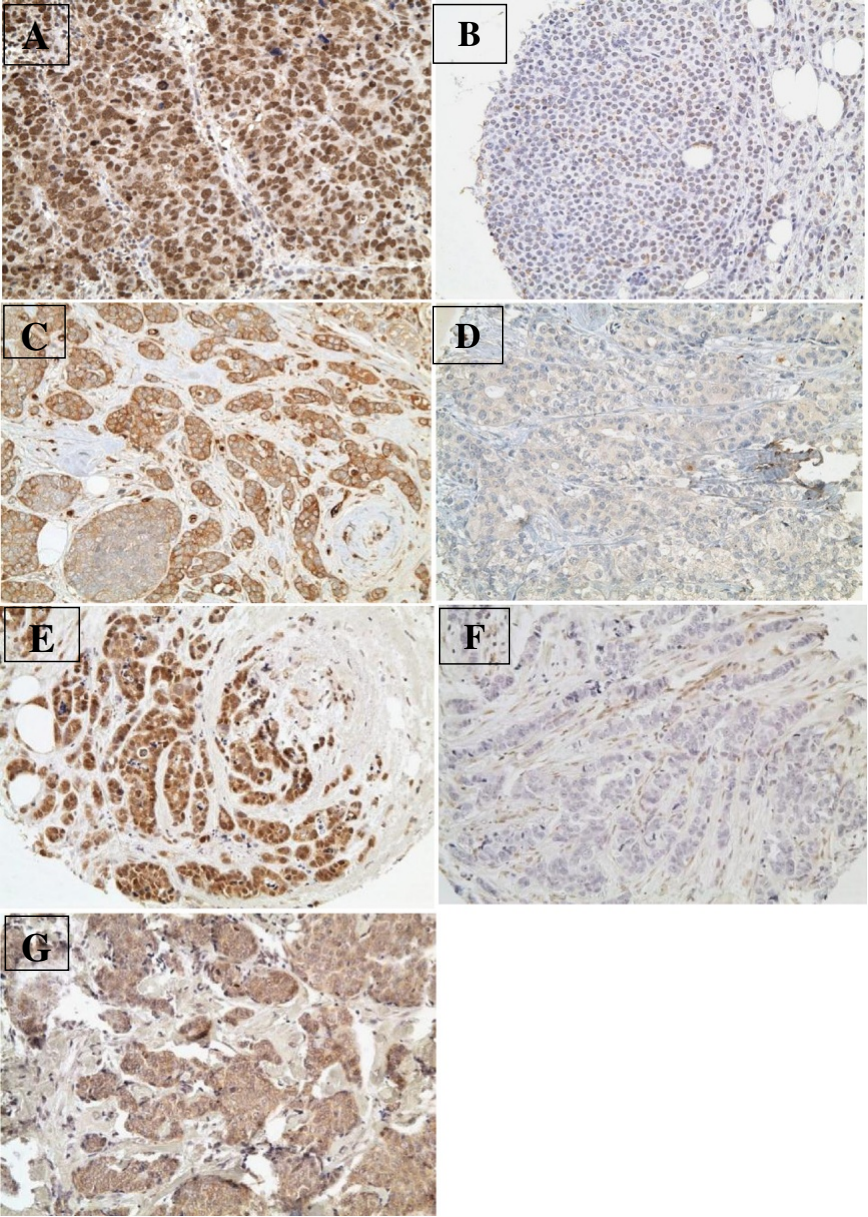
Representative images of immunohistochemical stainings showing tumor cells with high **a** and low **b** expression of TIF1γ, Tumor cells with high **c** and low **d** expression of TGFβ1, Tumor cells with high expression **e** of nuclear SMAD4, low expression of nuclear and cytoplasmic SMAD4 **f** and Tumor cells with high expression **g** of cytoplasmic SMAD4

Representative images of immunohistochemical stainings showing tumor cells with high and low expression of different markers are shown in Fig. 1. TIF1γ was high in 80 cases (35.9 %) (Fig. 1a) while 143 cases (64.1 %) showed low expression (Fig. 1b). TGFβ1 showed high expression in 62 (30.4 %) (Fig. 1c) while 142 cases (69.6 %) showed low expression (Fig. 1d).

Nuclear SMAD4 was high in 48 cases (27.7 %) (Fig. 1e) and low in 125 cases (72.3 %) while cytoplasmic SMAD4 was high in 91 cases (52.6 %) (Fig. 1g) and low in 82 cases (47.4 %). Low expression of both nuclear and cytoplasmic SMAD4 (SMAD4 loss) was detected in 70 patients (40.5 %) of the 173 patients (Fig. 1f). No correlation could be detected between the expression of TGFβ1, TIF1γ, and cytoplasmic or nuclear expression of SMAD4 (Additional file 1: Table S2).

### High TIF1γ expression and loss of SMAD4 are associated with poor prognostic factors

The correlations between clinical parameters and different predictive and prognostic factors with TGFβ1, TIF1γ and SMAD4 expression are shown in Table 1.

**Table 1:** Correlation between nuclear TIF1γ, cytoplasmic TGFβ1and SMAD4 expression with the clinico-pathologic parameters of breast cancer

Variable		TIF1γ low	TIF1γ high	P^a^	TGFβ1 low	TGFβ1 high	P^a^	SMAD4 low	SMAD4 high	P^a^
		No. (%)	No. (%)		No. (%)	No. (%)		No. (%)	No. (%)	
		143 (64.1)	80 (35.9)		142 (69.6)	62 (30.4)		70 (40.5)	103 (59.5)	
Age (Yr)	*-Mean (± SD)*	59.4(±12)	55.9(±13)	0.048^b^	57.6(±12)	59.3(±14)	0.37^b^	56.5(±12)	57.1 (±12)	0.74^b^
Age groups	*- ≤50y*	37 (26)	35 (44)	0.006	44 (31)	21 (34)	0.68	22 (31)	38 (37)	0.46
	*- >50y*	106 (74)	45 (56)		98 (69)	41 (66)		48 (69)	65 (63)	
Side	*-Right*	68 (48)	37 (46)	0.85	62 (44)	28 (45)	0.84	30 (43)	51 (49)	0.39
	*-Left*	75 (52)	43 (54)		80 (56)	34 (55)		40 (57)	52 (51)	
T. size	*- ≤2 cm*	80 (56)	35 (44)	0.08	73 (51)	30 (48)	0.69	31 (44)	51 (49)	0.49
	*- >2 cm*	63 (44)	45 (56)		69 (49)	32 (52)		39 (56)	52 (51)	
LN met	*-Negative*	85 (59)	41 (51)	0.24	79 (56)	35 (57)	0.91	36 (51)	55 (53)	0.79
	*-Positive*	58 (41)	39 (49)		63 (44)	27 (44)		34 (49)	48 (47)	
SBR grade	*-Gr 1*	39 (27)	6 (7)	<0.001	30 (21)	11 (18)	0.82	5 (7)	26 (25)	0.004
	*-Gr 2*	72 (50)	30 (38)		63 (44)	30 (48)		35 (50)	50 (49)	
	*-Gr 3*	32 (23)	44 (55)		49 (35)	21 (34)		30 (43)	27 (26)	
ER status	*-Negative*	13 (9)	15 (19)	0.035	24 (17)	6 (10)	0.17	13 (19)	7 (7)	0.02
	*-Positive*	129 (91)	64 (81)		116 (83)	56 (90)		56 (81)	95 (93)	
PR status	*-Negative*	28 (20)	22 (28)	0.18	38 (27)	11 (18)	0.14	18 (26)	21 (21)	0.40
	*-Positive*	113 (80)	57 (72)		101 (73)	51 (82)		51 (74)	81 (79)	
Her 2 status	*-Negative*	126 (91)	69 (87)	0.35	125 (91)	54 (89)	0.66	60 (90)	90 (89)	0.93
	*-Over-expressed*	12 (9)	10 (13)		13 (9)	7 (11)		7 (10)	11 (11)	
Breast cancer subtype	*-Luminal*	127 (91)	64 (81)	0.12^c^	114 (83)	55 (90)	0.31^c^	54 (81)	94 (93)	0.05^c^
	*-Her2 rich*	2 (1)	3 (4)		4 (3)	2 (3)		2 (3)	1 (1)	
	*-Basal*	11 (8)	12 (15)		20 (14)	4 (7)		11 (16)	6 (6)	
(Neo)/ Adjuv. Hormonal ttt	*-Tamoxifen*	101 (89)	54 (87)	0.89^c^	95 (92)	44 (83)	0.20^c^	48 (89)	75 (94)	0.48^c^
	*-AI*	4 (4)	3 (5)		3 (3)	2 (4)		1 (2)	1 (1)	
	*-Tamoxifen + AI*	8 (7)	5 (8)		5 (5)	7 (13)		5 (9)	4 (5)	
(Neo)/ Adjuv. chemotherapy	*-Anthra.*	52 (85)	40 (82)	0.61	58 (76)	25 (83)	0.59	34 (74)	44 (80)	0.48^c^
	*only*	9 (15)	9 (18)		16 (21)	5 (17)	^c^	11 (24)	11 (20)	
	*-Anthra. +*							1 (2)	0 (0)	
	*Taxane*	0 (0)	0 (0)		2 (3)	0 (0)				
	*-Others*									

^*a*^Correlations tested by Pearson’s Chi square test (2sided) unless otherwise specified

^b^ Difference between means by Student’s T test

^c^ Fisher’s exact test

TGFβ1 expression was not correlated to any of the traditional prognostic markers such as age, tumor size, SBR grade, axillary lymph node metastasis, ER, PR, and HER2 status. Interestingly, high TIF1γ expression was associated with younger age (p = 0.006), higher SBR grade (p < 0.001), ER negativity (p = 0.035), and a tendency towards larger tumors (p = 0.08).

Loss of SMAD4 expression (cytoplasmic or nuclear) was associated with higher tumor SBR grade (p = 0.004) and more ER negativity (p = 0.02), while the nuclear localization of SMAD4 was not associated with any of the clinico-pathological parameters.

### High expression of TGFβ1 and TIF1γ are associated with poor clinical outcome

TGFβ1 expression was associated with more deaths (30.4 % of patients with high expression died versus 17.6 % in patients with low expression, p = 0.04), more disease recurrences (35.5 % of patients with high expression versus 17.6 % in patients with low expression, p = 0.005), and more metastatic relapses (33.9 % of patients with high expression versus 14.1 % in patients with low expression, p = 0.001).

TIF1γ expression (as a single marker) also showed similar trends towards increased number of deaths and distant metastases however, not reaching statistical significance. The loss of SMAD4 expression did not correlate with more deaths or relapses. Death and relapse events in correlation with the biomarkers expression are shown in Table 2.

**Table 2:** Death, relapse, and metastatic events in correlation with TIF1γ and TGFβ1 expression

Events		TIF1γ low	TIF1γ high	P^*^	TGFβ1 low	TGFβ1 high	P^*^	SMAD4 low	SMAD4 high	P^*^
		No. (%)	No. (%)		No. (%)	No. (%)		No. (%)	No. (%)	
		143 (64.1)	80 (35.9)		142 (69.6)	62 (30.4)		70 (40.5)	103 (59.5)	
Death	*-Alive*	115 (80)	57 (71)	0.11	117 (82)	43 (69)	0.04	56 (80)	82 (80)	0.95
	*-Dead*	28 (20)	23 (29)		25 (18)	19 (31)		14 (20)	21 (20)	
Recurrence	*-No*	113 (79)	59 (74)	0.37	117 (82)	40 (65)	0.005	56 (80)	78 (76)	0.51
	*-Yes*	30 (21)	21 (26)		25 (18)	22 (35)		14 (20)	25 (24)	
DistantMetast.	*-No*	118 (83)	60 (75)	0.18	122 (86)	41 (66)	0.001	58 (83)	81 (79)	0.49
	*-Yes*	25 (17)	20 (25)		20 (14)	21 (34)		12 (17)	22 (21)	

^*^Correlations tested by Pearson’s Chi square test

Of interest, the pattern of distant metastases was correlated with the 3 biomarkers expression. For example among the 10 patients having bone only metastases, 9 of them expressed SMAD4 (in the nucleus or cytoplasm) while only one patient had no SMAD4 expression (p = 0.050). On the other hand, patients co-expressing TGFβ1 and TIF1γ had more chance of visceral metastases than the rest of the patients population (24.2 % versus 7.3 %, p = 0.002), while such tendency did not appear for bone metastases (p = 0.74).

**Fig. 2:**
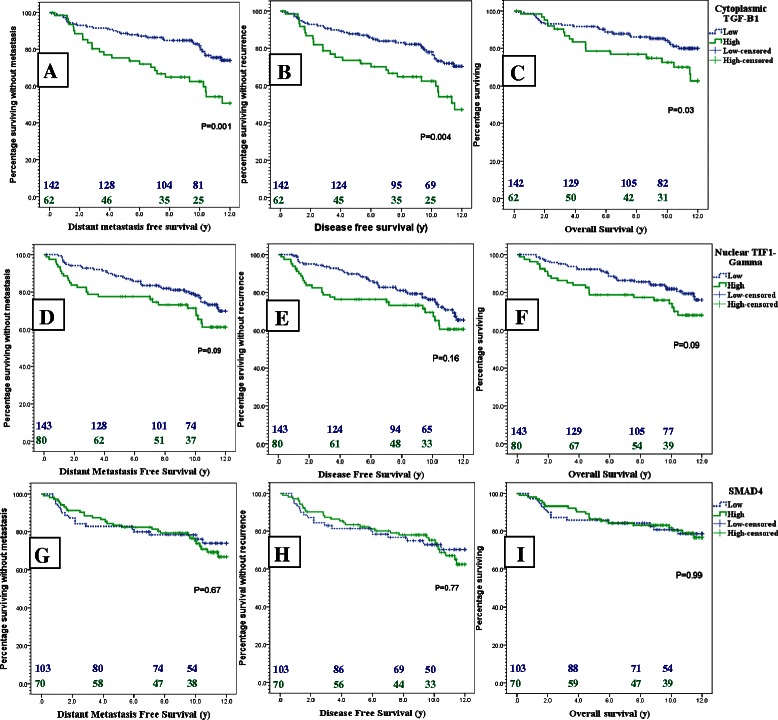
Kaplan Meier plots for the impact of low (Dotted blue) versus high (green) expression of TGFβ1 **a**, **b**, **c**, TIF1γ **d**, **e**, **f** and SMAD4 **g**, **h**, **i** on DMFS **a**, **d**, **g**, DFS **b**, **e**, **h** and OS **c**, **f**, **i** in breast cancer patients

Regarding the patients’ survival, DMFS was shorter in patients highly expressing TGFβ1 than those with low expression with an 8y DMFS rate of 62.5 % (95 % CI: 46.5-78.5 %) compared to 83.2 % (95 % CI: 76–90.4 %) respectively (p = 0.001). DFS was also shortened in high TGFβ1 patients with an 8y DFS rate of 63.5 % (95 % CI: 46.5-78.5 %) versus 82.1 % (95 % CI: 74.7-89.4 %) in the low expression group (p = 0.004). OS was worse in cases with high TGFβ1 expression with an 8y OS rate of 75 % (95 % CI: 61.9-88.1 %) versus 84.7 % (95 % CI: 77.8-91.6 %) in the TGFβ1 low group (p = 0.03). Fig. 2 shows the Kaplan Meier’s curves for DMFS, DFS and OS according to different biomarker expression.

According to the Cox proportional hazard model high TGFβ1 expression almost doubled the risk of developing distant metastases (HR = 2.28, 95 % CI: 1.4 to 3.8, p = 0.002), and death by 80 % (HR = 1.89, 95 % CI: 1.04 to 3.43, p = 0.037).

Regarding the effect of the classical prognostic factors, DMFS was shorter with tumors larger than 2 cm (HR = 2.61, 95 % CI: 1.59 to 4.30, p = 0.0002), axillary LN involvement (HR = 2.02, 95 % CI: 1.26 to 3.26, p = 0.004), High SBR grade (HR = 2.61, 95 % CI: 1.64 to 4.16, p = 0.00006), ER negative (HR = 2.36, 95 % CI: 1.35 to 4.12, p = 0.003), and PR negative tumors (HR = 1.72, 95 % CI: 1.042 to 2.85, p = 0.03).

Importantly, in the multivariate analysis, when adjusted to tumor size, lymph node positivity, SBR grade, ER and PR status, TGFβ1 expression was still an independent predictor for distant metastasis (HR = 2.56, 95 % CI: 1.5 to 4.3, p < 0.0001) and death (HR = 2.06; 95 % CI: 1.13 to 3.75, p = 0.018). In addition to TGFβ1, large tumor size (HR = 2.10, 95 % CI: 1.25 to 3.49, p = 0.005) and high SBR grade (HR = 1.97, 95 % CI: 1.16 to 3.36, p = 0.013) were the only factors that independently predicted shorter DMFS in the multivariate model.

For TIF1γ expression there was a tendency towards increased risk of metastasis (HR = 1.54; 95 % CI: 0.93 to 2.54, p = 0.09), and death (HR = 1.6; 95 % CI: 0.9 to 2.8, p = 0.09) in cases of high TIF1γ expression compared to cases with low expression.

SMAD4 expression (including nuclear localization) showed no prognostic significance for DMFS, DFS or OS.

### Prognostic significance of TGFβ1 is limited to more advanced tumor stages

Interestingly, the effect of TGFβ1 expression on the outcome of breast cancer was observed to be limited to tumors with higher T and N stages. For example, in tumors smaller than 20 mm in the maximal dimension, the 8 years DFS rate was 86.7 % (95 % CI: 76.7-96.7 %) in patients with low TGFβ1 expression versus 86.3 % (95 % CI: 73.2-99.3 %) in patients with high TGFβ1 (p = 0.91). In larger tumors however, the 8 years DFS rate was 79.2 % (95 % CI: 64.1-91.3 %) versus 43.7 % (95 % CI: 16.7-70.7 %) in patients with low versus high TGFβ1 expression respectively (p = 0.0003).

**Fig. 3:**
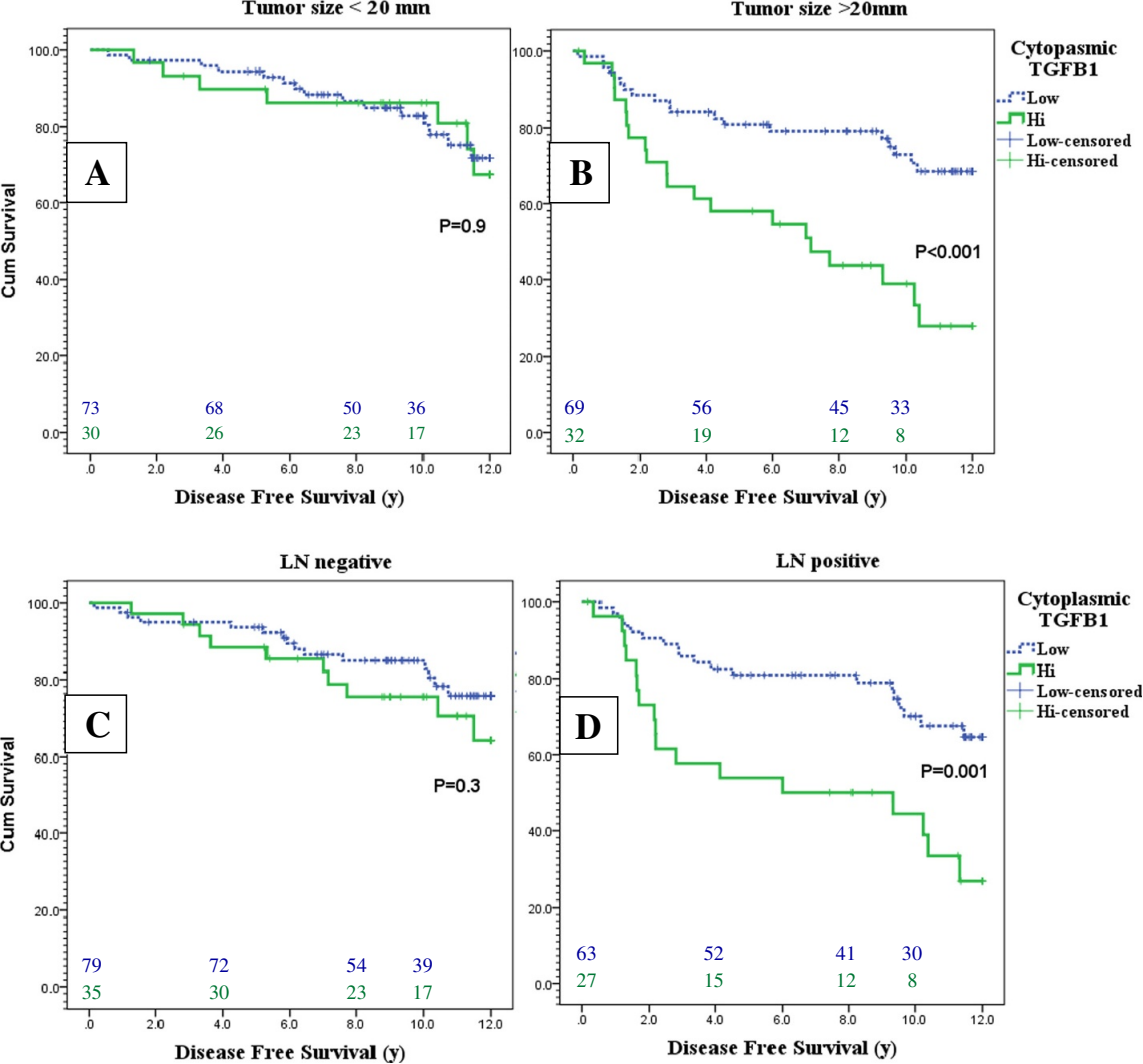
Kaplan Meier curves for DFS of TGFβ1 low (Dotted blue) versus high (green) expression in patients with tumors ≤ 20 mm **a**, > 20 mm **b**, N0-1mic **c** and N1a-3 **d**

Similarly, in patients with no axillary LN metastasis the 8 years DFS rate was 84.9 % (95 % CI: 74.9-94.9 %) versus 75.5 % (95 % CI: 57.6-93.4 %) in patients with low versus high TGFβ1 expression respectively (p = 0.31). On the other hand, in patients with axillary LN metastasis, the 8 years DFS rate was 80.9 % (95 % CI: 68.8-93.0 %) versus 50.0 % (95 % CI: 20.8 %-79.2 %) in patients with low versus high TGFβ1 expression respectively (p = 0.001). Kaplan Meier curves for DFS in TGFβ1 high versus low expression in early (T size ≤ 20 mm and LN negative) and advanced (T size > 20 mm and LN positive) stages are presented in Fig. 3.

### TIF1γ restricts the prognostic significance of TGFβ1

**Fig. 4:**
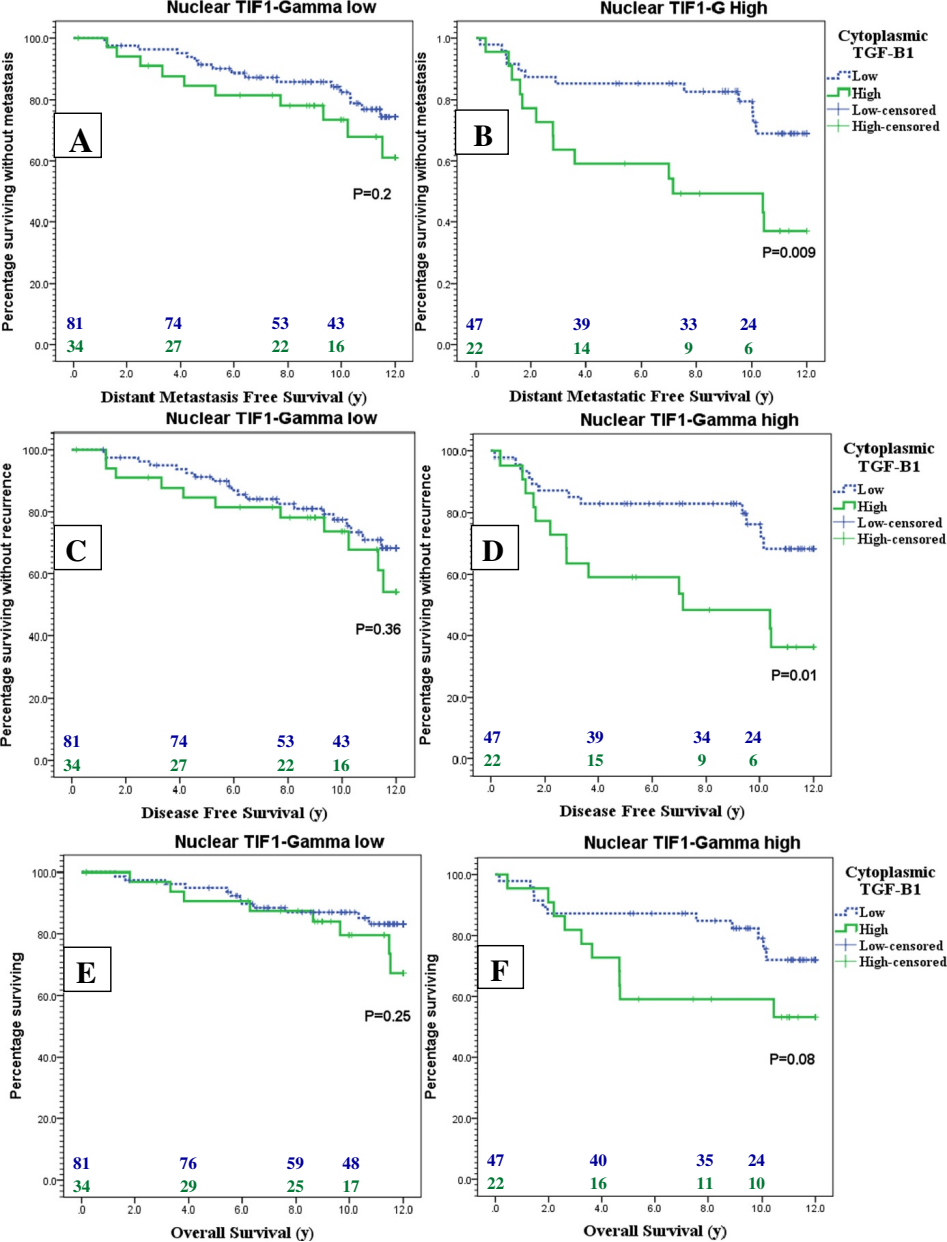
Kaplan Meier plots for DMFS **a**, **b**, DFS **c**,**d** and OS **e**, **f** of TGFβ1 high (green) versus low expression (Dotted blue) in TIF1γ low patients **a**, **c**, **e** and TIF1γ high patients **b**, **d**, **f**

Strikingly, the ability of TGFβ1 expression to predict the poor outcome in breast cancer patients was restricted to TIF1γ expressing tumors. For example, in the patient population with TIF1γ high expression the 8y DMFS rate was 49.2 % (95 % CI: 16.3-80.8 %) in patients with high TGFβ1 expression versus 82.7 % (95 % CI: 79.2-94.2 %) in patients with TGFβ1 low expression (p = 0.009). On the other hand, in the patient population with TIF1γ low expression the 8y DMFS rate was 78.1 % (95 % CI: 65.9-90.2 %) versus 85.9 % (95 % CI: 75.5-96.3 %) in high versus low TGFβ1 respectively (p = 0.2). The same pattern of difference was also observed in DFS and OS as shown in Kaplan Meier curves in Fig. 4.

**Fig. 5:**
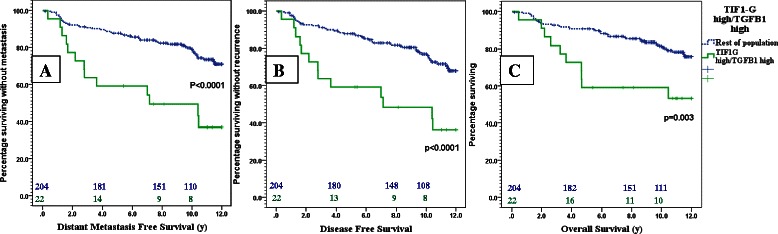
Kaplan Meier curves showing the DMFS **a**, DFS **b** and OS **c** of the TGFβ1-high/TIF1γ-high patients (green) versus the rest of patient population (Dotted blue)

Moreover, the subgroup of patients expressing both TGFβ1 and TIF1γ had the poorest prognosis when compared to the rest of the patients' population. The 8 years DMFS, DFS and OS rates were 49.2 % (95 % CI: 16.3-80.8 %), 48.3 % (95 % CI: 15.2-75.3 %) and 59.1 % (95 % CI: 29.8-88.4 %) in the TGFβ1-high/TIF1γ-high subgroup versus 82.4 % (95 % CI: 75.2-89.8 %), 81.8 % (95 % CI: 74.4-89.2 %) and 85.3 % (95 % CI: 91.2-79.4 %) in the rest of the population (P < 0.001, <0.001 and =0.003 respectively). Kaplan Meier curves of DMFS, DFS and OS of this subgroup are shown in Fig. 5.

According to Cox regression model the co-expression of TGFβ1 and TIF1γ increased the risk of metastases (HR = 3.2; 95 % CI: 1.7 to 5.9, p < 0.0001), any recurrence (HR = 3.02; 95 % CI: 1.6 to 5.6, p < 0.0001) and death from any cause (HR = 2.7; 95 % CI: 1.4 to 5.4, p = 0.005). In the multivariate analysis, and when adjusted to age, SBR grade, tumor size, and lymph node invasion the co-expression of TGFβ1 and TIF1γ remained an independent poor prognostic factor that predicted metastasis, any recurrence and death from any cause.

### TIF1γ strongly predicts relapse and death in patients with SMAD4 loss

**Fig. 6:**
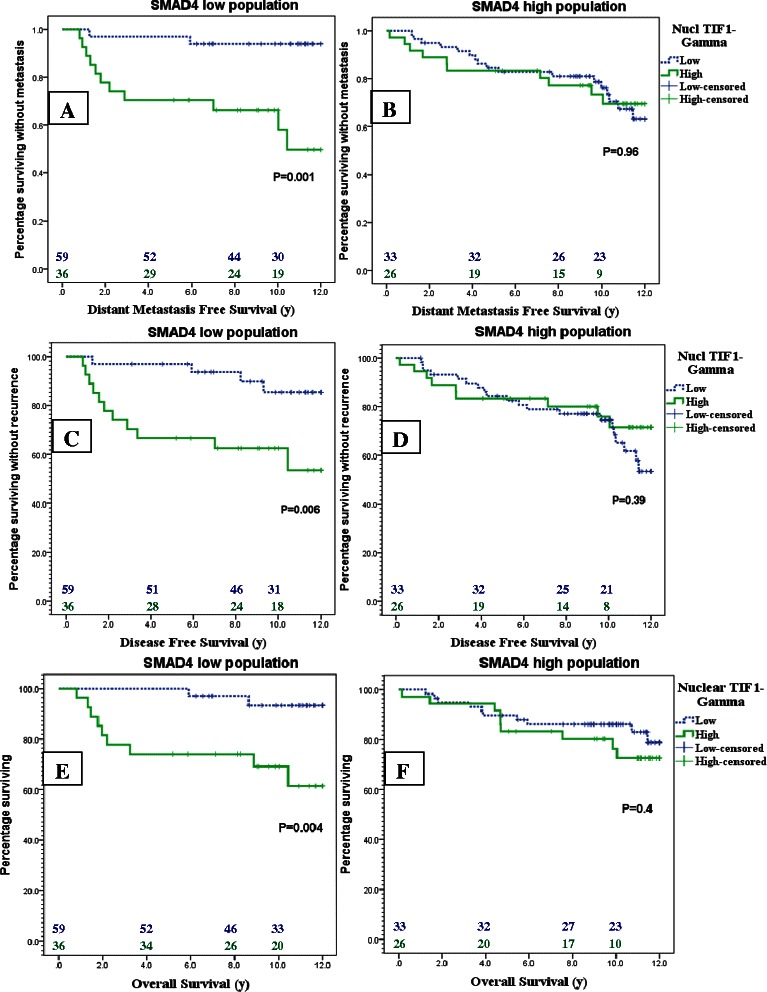
Kaplan Meier curves of DMFS **a**, **b**, DFS **c**, **d** and OS **e**, **f** curves for TIF1γ high (green) versus low expression (Dotted blue) in cases of SMAD4 loss **a**, **c**, **e** and SMAD4 expression **b**, **d**, **f**

Low expression of SMAD4 appeared to manipulate the outcome of patients with TIF1γ high versus low expression. In patients with SMAD4-low tumors, TIF1γ expression strongly predicted metastasis, any cancer recurrence and death, while in patients with SMAD4 high expression, the TIF1γ expression did not show any prognostic value. In patients with SMAD4 loss, the 8 years DMFS rate was 66.2 % (95 % CI: 41.2-91.2 %) in patients with high TIF1γ versus 93.9 % (95 % CI: 82.9-100 %) in patients with low TIF1γ (p = 0.001), while it was 77.2 % (95 % CI: 60.0-94.4 %) versus 81.0 % (95 % CI: 70.5-91.5 %) respectively (p = 0.96) in the SMAD4 expressing group. Similarly, DFS and OS showed the same difference only in patients with low SMAD4 expression (p = 0.006 and 0.004 respectively) while such differences were absent with high SMAD expression (p = 0.39 and 0.40 respectively). Figure 6 shows Kaplan Meier curves for DMFS, DFS and OS in patients with TIF1γ high versus low expression according to SMAD4 status of the tumors.

## Discussion

In our present work we found a definitive poor outcome in patients whose primary breast tumors showed higher expression of cytoplasmic TGFβ1. Those patients had an increased risk of metastasis and death and this risk was independent from the other major prognostic factors such as tumor size, LN involvement, SBR grade and hormone receptor status. The poor outcome in those patients was restricted to patients with larger tumors (more than 2 cm) and axillary lymph node metastasis (N1a-N3), an observation that might be explained by the dual role of TGFβ signaling in different stages of carcinogenesis.

Our results are in agreement with the work of Desruisseau et al. who reported that a high TGFβ1 protein level measured by enzyme-immunoassay in breast cancer tissue was an independent poor prognostic marker for disease free survival [21]. Richardsen et al. also reported that high stromal expression of TGFβ in breast cancer areas was associated with increased mortality. [22]. However, other studies showed opposing results with better DFS and OS in patients with high TGFβ1 and TGFβ receptor type II expression [18] and lower recurrence rates with patients expressing TGFβ1 and pSMAD2/3 [20].

Our data may help to provide explanations for some of the discrepancies in the results of previous studies testing TGFβ1/SMAD4 pathway biomarkers expression. Such discrepancies might be explained by the different effect of TGFβ1 expression in early versus advanced tumor stages, the heterogeneous population included in such retrospective trials and the crosstalk between TGFβ signaling and other pathways. Indeed in our study, we also observed a significant interaction between TGFβ1, TIF1γ expression and the prognosis of breast cancer patients, an interaction that was not investigated in previous studies. The subgroup of patients expressing both TGFβ1 and TIF1γ showed the poorest outcome compared to the rest of the patient population. Finally, the different scoring systems for biomarker staining in those studies may account for this diversity, putting into account what was suggested by Bierie et al. that gain or complete loss of TGFβ signaling may result in gene expression signatures correlated with poor prognosis in breast cancer [27].

On the other hand, TIF1γ expression showed tendency towards poor outcome in breast cancer patients. That tendency became significant when combined with TGFβ1 expression and SMAD4 loss. To our knowledge this is the first study to report such interactions which might be unexpected in view of the available data suggesting a role for TIF1γ in inhibiting epithelial to mesenchymal transition (EMT) through repression of SMAD4 activity and hence interfering with tumor progression and metastasis [11]. This tumor inhibitory role was also observed against murine and human tumors including pancreatic, hepatocellular carcinomas and leukemia [14–16]. However, our findings are in agreement with the observation by Jain et al. that overexpression of TIF1γ was associated with colorectal cancer incidence and poor prognosis [17].

Regarding the value of SMAD4 expression, we did not find any correlation between SMAD4 expression and any of the clinico-pathological parameters of breast cancer. We did not find also any prognostic significance of either nuclear localization or total loss of SMAD4 expression. This may be concordant with some studies that tested the effect of SMAD4 expression in operable breast cancer [28].

On the other hand 2 important observations were found regarding SMAD4 expression. The first is the presence of a significant interaction between TIF1γ and SMAD4 that alters the patients’ outcome regarding distant metastases and overall survival. We have shown that TIF1γ predicts poor outcome of breast cancer patients only in cases with SMAD4 loss, while in breast cancer patients whose tumors expressed SMAD4 no difference in survival was detected. The idea of combining SMAD4 loss with other biomarkers expression was tackled in a study by De Kruijf et al. combining SMAD4 loss with the expression of TGFβ type I & II receptors that could identify a subgroup of stage I to III breast cancer carrying the poorest outcome [29].

The second observation was the strong association between SMAD4 expression and the pattern of relapse of breast cancer. Almost all patients with only bone metastasis expressed SMAD4 either in the cytoplasm or the nucleus. Such observation are in accordance with previous studies, in xenograft models and cell lines, showing that SMAD4 signaling is needed for the formation of osteolytic bone metastases, an observation that was confirmed by the knockdown of SMAD4 in breast cancer cells which could protect against bone metastasis in nude mice with significantly increasing metastasis free survival [30, 31].

Surprisingly, we found a poor outcome in patients with co-expression of TIF1γ with either TGFβ1 high or SMAD4 low. A possible hypothesis is that TIF1γ competes with SMAD4 turning off the SMAD4 dependent TGFβ signaling. Such dual effect of the TGF-β signaling might be influenced by the varying TIF1γ/Smad4 ratios resulting in the modulation of the transcriptional signal induced by TGFβ as suggested by Andrieux et al. [32]. We cannot also exclude that tumor-cell-derived TGFβ acts on the surrounding tissue in a paracrine manner instead of an autocrine signaling in the tumor cells themselves. Interactions between tumor cells and cancer-associated fibroblasts (CAFs) in the tumor microenvironment significantly influence cancer growth and metastasis, and TGFβ is known to be critical for CAF activation and elaboration of a pro-tumorigenic microenvironment [6].

Our work, however is limited by its retrospective nature, the use of TMA sections that bear only cores of the whole tumor, the absence of a validation of our results in an independent cohort (preferably in multiple centers) in addition to the heterogeneity of the patient cohort regarding the adjuvant treatment received which may bias the results. Larger prospective biomarker-oriented studies are needed to further clarify the missing pieces of the TGF pathway story.

## Conclusion

Our present work clearly concludes that there is a crosstalk between the TIF1γ and the TGFβ1/SMAD4 pathway that can predict poorer outcome in operable breast cancer patients. Such prediction of poor outcome was more evident in tumors with higher stages. We could also conclude that the value of TGFβ1, SMAD4 and TIF1γ expression in breast cancer should not be considered individually but instead combined to serve as an effective prognostic tool for breast cancer. The value of such information is of utmost importance with the introduction of new targeted agents against the TGFβ axis [33–35]. The upcoming trials testing those agents in breast cancer represent a golden opportunity for clearly understanding the impact of this pathway on the disease outcome in addition to finding biomarkers that could predict benefit of such drugs.

## Additional file

Additional file 1: Table S1. Clinicopathological characteristics and biomarker expression in the tested patients’ population (248 patients). **Table S2**: Absence of correlations between expression of TGFβ1, TIF1Ɣ, cytoplasmic and nuclear SMAD4. *Correlations tested by Pearson’s Chi square test. **Figure S1:** A flowchart of the whole population and subsets tested for different biomarkers.
