# Supplementary material for: TIF1γ interferes with TGFβ1/SMAD4 signaling to promote poor outcome in operable breast cancer patients
Source: BMC Cancer. 2015 Jun 4;15:453. doi: 10.1186/s12885-015-1471-y (PMC4453047; doi:10.1186/s12885-015-1471-y)
Supplement: Additional file 1: Table S1. — Clinicopathological characteristics and biomarker expression in the tested patients’ population (248 patients). Table S2: Absence of correlations between expression of TGFβ1, TIF1Ɣ, cytoplasmic and nuclear SMAD4. *Correlations tested by Pearson’s Chi square test. Figure S1: A flowchart of the whole population and subsets tested for different biomarkers. [file 12885_2015_1471_MOESM1_ESM.pdf]

Characteristic		Number	percent
**Age group**	*≤ 50 years*	78	31.5%
	>50 years	170	68.5%
**Tumor size**	*≤2cm*	126	50.8%
	>2cm	122	49.2%
**Axillary LN**	*No*	136	54.8%
**metastasis**	*Yes*	112	45.2%
**SBR grade**	*I*	48	19.4%
	II	116	46.8%
	III	84	33.9%
**ER status**	*Negative*	36	14.6%
	Positive	210	85.4%
	Missing	2	
**PR status**	*Negative*	60	24.5%
	Positive	185	75.5%
	Missing	3	
**Her2 status**	*Negative*	217	90%
	Positive	24	10%
	Missing	7	
**Adjuvant Hormonal**	*No*	56	22.6%
**treatment**	*Yes*	192	77.4%
**Adjuvant (or**	*No*	108	43.5%
**neoadj)**	*Yes*	140	56.5%
**chemotherapy**			
**TIF1γ**	*Low*	143	64.1%
	High	80	35.9%
	Missing	25	
**TGFβ1**	*Low*	142	69.6%
	High	62	30.4%
	Missing	44	
**Nuclear SMAD4**	*Low*	125	72.3%
	High	48	27.7%
	Missing	75	
**Cytoplasmic**	*Low*	82	47.4%
**SMAD4**	*High*	91	52.6%
	Missing	75	

Table S1: Clinicopathological characteristics and biomarker expression in the tested patients' population (248 patients)

Marker		TIF1γ low		TIF1γ high		P value*	TGFβ1 low		TGFβ1 high		P value*
		No.	(%)	No.	(%)		No.	(%)	No.	(%)	
**Nuclear**	Low	66	(71.7%)	46	(73%)	P=0.86	85	(75.2%)	27	(67.5%)	P=0.34
**SMAD 4**	High	26	(28.3%)	17	(27%)		28	(24.8%)	13	(32.5%)	
**Cytopl.**	Low	40	(43.5%)	31	(49.2%)	P=0.48	50	(44.2%)	19	(47.5%)	P=0.7
**SMAD4**	High	52	(56.5%)	32	(50.8%)		63	(55.8%)	21	(52.5%)	
**SMAD**	Yes	33	(35.9%)	27	(42.9%)	P=0.38	45	(39.8%)	15	(37.5%)	P=0.8
**loss**	No	59	(64.1%)	36	(57.1%)		68	(60.2%)	25	(62.5%)	
**TGFβ1**	Low	81	(70.4%)	47	(68.1%)	P=0.74					
	high	34	(29.6%)	22	(31.9%)						

Table S2: Absence of correlations between expression of TGFβ1, TIF1γ, cytoplasmic and nuclear SMAD4.

[*]Correlations tested by Pearson's Chi square test

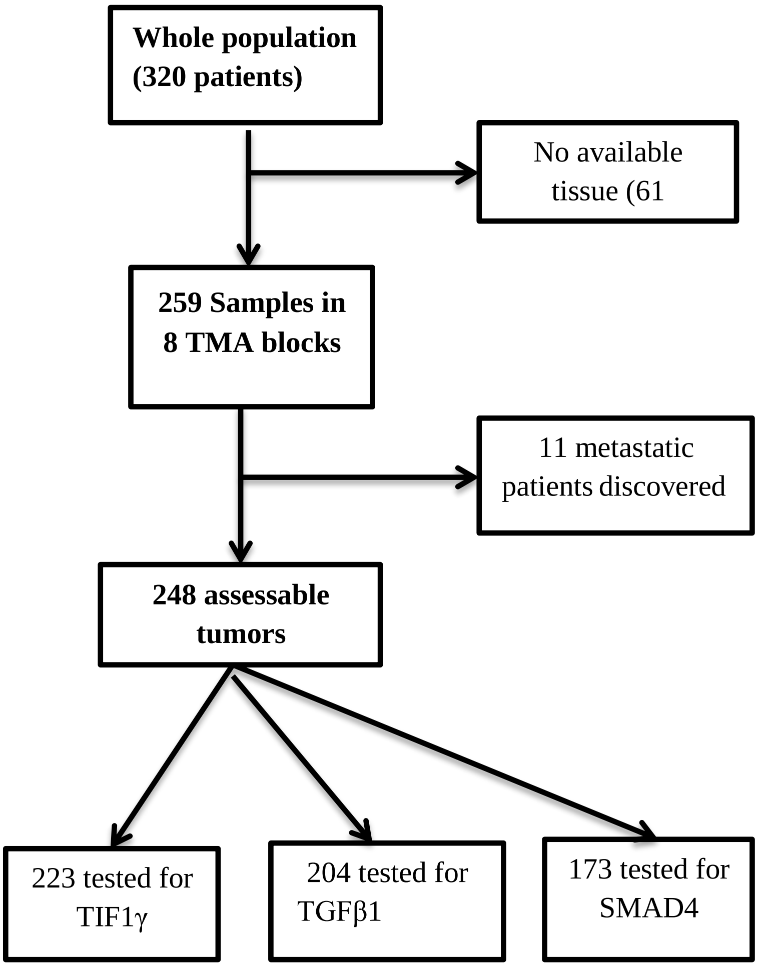

Figure S1: A flowchart of the whole population and subsets tested for different biomarkers
